# Glycemic load is associated with HDL cholesterol but not with the other components and prevalence of metabolic syndrome in the third National Health and Nutrition Examination Survey, 1988–1994

**DOI:** 10.1186/1755-7682-2-3

**Published:** 2009-01-13

**Authors:** Amy Culberson, Mohammad R Kafai, Vijay Ganji

**Affiliations:** 1 School Nutrition, McDuffie County Schools, Thomson, GA 30824, USA; 2 Department of Mathematics, San Francisco State University, San Francisco, CA 94132, USA; 3 Division of Nutrition, School of Health Professions, College of Health and Human Sciences, Georgia State University, Atlanta, GA 30303, USA

## Abstract

**Background:**

Carbohydrate quality and quantity may affect the risk for cardiovascular diseases (CVD) and type-2 diabetes mellitus. Glycemic load (GL) is a mathematical concept based on carbohydrate quality and quantity. GL is a product of glycemic index (GI) and the carbohydrate content of a food item divided by 100.

**Objective:**

In this study, the association between GL and components and prevalence of metabolic syndrome was investigated in a representative sample survey of US residents utilizing the data reported in the third National Health and Nutrition Examination Survey (*n *= 5011).

**Methods:**

Metabolic syndrome was defined according to the criteria established by the Adult Treatment Panel III. Multivariate-adjusted means for waist circumference, triacylglycerol, systolic and diastolic blood pressures, blood glucose, and HDL cholesterol were determined according to the energy-adjusted GL intake quartiles using regression models.

**Results:**

In all subjects and in men, high GL was associated with low HDL-cholesterol concentrations in multivariate-adjusted analysis (*P *for trend < 0.01). However, no association was observed between GL and any of the individual components of metabolic syndrome in women. Also, no association was observed between energy-adjusted GL and prevalence of metabolic syndrome in both men (*P *for trend < 0.21) and women (*P *for trend < 0.09) in the multivariate-adjusted logistic regression analysis.

**Conclusion:**

It is likely that the diets low in GL may mitigate the risk for CVD through HDL cholesterol.

## Background

Metabolic syndrome is characterized by central adiposity, dyslipidemia, impaired glucose tolerance, insulin resistance, and hypertension. Metabolic syndrome affects approximately 27% of the U.S. population [1]. A low-fat diet is currently recommended for the treatment of metabolic syndrome in order to facilitate weight loss [2]. However, evidence from epidemiological studies does not show a consistent relation between the intake of dietary fat and adiposity [3]. Energy from fat is often replaced with energy from carbohydrates [4]. A high carbohydrate diet, with poor regard for carbohydrate quality, may produce the biochemical features of metabolic syndrome [5]. High carbohydrate diets can adversely affect the lipid profile and glucose tolerance, worsening the metabolic abnormalities in those with or predisposed to metabolic syndrome [6,7].

The glycemic index (GI) is based on the glycemic response to a fixed amount of carbohydrate [8]. GI is an indicator of the quality of dietary carbohydrate [9]. Diets high in GI are associated with greater fluctuations in blood glucose and insulin concentrations compared to those diets low in GI [10]. The glycemic load (GL) is a mathematical model and is defined as the product of the available total carbohydrate content of a food item in grams and the corresponding GI of that food, divided by 100 [9]. Thus, GL represents both quality and quantity of dietary carbohydrate. Foods with a high GL elicit greater glycemic and insulinemic responses [10].

Individuals with metabolic syndrome are at increased risk of developing cardiovascular disease (CVD) and type-2 diabetes [11,12]. Epidemiological evidence suggests that the diets high in GI and GL are associated with increased risk for CVD [13] and type-2 diabetes [9]. Little research has been conducted on the association between GL and indices and prevalence of metabolic syndrome. One study examined the relation of GL with the prevalence of metabolic syndrome using data from the Framingham Study Cohort [14]. However, the population used in that study was predominantly white. The risk of developing related outcomes of metabolic syndrome increases with multiple risk factors [15]. It is therefore important to study GL and all the risk factors of metabolic syndrome together. The aim of this study was to investigate the association between dietary GL and indices and prevalence of metabolic syndrome using the data from a representative survey of the US population.

## Methods

### Survey design and study sample

The third National Health and Nutrition Examination Survey, 1988–1994 (NHANES III) was conducted by the National Center for Health Statistics of the Centers for Disease Control and Prevention and was designed to collect data on the health and nutritional status of US residents [16]. Data used for the analysis were derived from public use data files released by the National Technical Information Services, Springfield, VA [17]. NHANES III was a complex, stratified, probability sample survey of non-institutionalized individuals over the age of two months. Data were collected on demographics, socioeconomic status, physical and health conditions, biochemical constituents in blood and urine, anthropometric measurements, and dietary intake. Individuals with diabetes, pregnant and lactating women, individuals with missing data for food frequency, indices of metabolic syndrome and the covariates used for analysis, individuals who fasted <9 hours, and individuals whose dietary records were coded as unreliable were excluded from the data analysis. After applying the aforementioned exclusion criteria, the final study sample consisted of 5011 individuals (men, 3047; women, 1964).

### Measurements

Blood was collected by venipuncture and processed according to standard protocol [16]. Serum total cholesterol was measured enzymatically (Hitachi 704 Analyzer; Boehringer Mannheim Diagnostics, Indianapolis, IN) and serum triacylglycerol was measured following hydrolyzation to glycerol. Serum HDL cholesterol was measured following the precipitation of other lipoproteins with a manganese chloride-heparin solution. All lipoproteins were analyzed at the John Hopkins University Lipoprotein Analytical Laboratory, Baltimore, MD. Blood glucose was determined through an enzymatic reaction (Cobas Mira assay: Roche, Basel, Switzerland) at the University of Missouri-Columbia School of Medicine, Columbia, MO. Detailed analytical methodology has been described elsewhere [16].

Blood pressure was measured using a mercury sphygmomanometer (W A Baum Co, Inc, Copiague, NY) according to the standard protocol recommended by the American Heart Association [18]. The mean of 3–6 measurements was used. Waist circumference was measured to the nearest 0.1 cm. Body mass index (BMI) was computed from weight and height measurements. Poverty income ratio was used to define the socioeconomic status. A poverty income ratio < 1.0 is considered below the poverty level. Participants who answered 'yes' to the present smoking status question were treated as current smokers. Participants who answered 'yes' to the questions "have you taken vitamins/minerals and prescription medicine in the past month?" were treated as supplement users and medicine users, respectively. Alcohol intake data were collected for participants >17 y. Subjects were asked to report their consumption of beer, wine, and hard liquor over the past month prior to the survey. One drink of alcohol was described as 360 mL of beer, 120 mL of wine, or 30 mL of hard liquor. Total alcohol intake (drinks/month) was calculated by summing the drinks of beer, wine, and hard liquor.

### Dietary assessment and calculation of GL

In NHANES III, food intake data were collected using an 80-item qualitative food frequency questionnaire (FFQ) and one 24-hour food recall. FFQ was designed to provide qualitative information about the usual intake of individuals >17 y. Participants were asked how often they consumed a particular food or beverage over the past month prior to the survey. Food intake data were standardized to number of times consumed/month. Food recall data were collected by using an automated, microcomputer-based dietary interview and coding system. Participants reported all food and beverages consumed for the previous 24-hour time period. Nutrient composition of foods recalled was based on the United States Department of Agriculture Survey Nutrient Database [19].

Dietary intake data from the FFQ were used to calculate dietary GL. Previously, Ford and Liu [20] calculated GL and GI from the dietary intakes collected utilizing the FFQ in the NHANES III. If the frequency of consumption was reported as two, it was assumed that the participant consumed two portions. The carbohydrate content of foods consumed was determined using standard portion sizes from the USDA food composition tables [21]. GL value was calculated for each participant by multiplying the carbohydrate content in grams in one serving of a food item by the corresponding GI of that food divided by 100. This value was multiplied by the food frequency data of that food item to derive the GL. The individual GL values for each food item are summed to derive the total daily GL consumed. Dietary GL intake was determined according the formula given below.

$$\text{GL}=\sum_{i=1}^{n}({\text{GI}}_{i}{\text{ X CHO}}_{i}){\text{F}}_{i}/100$$

In the above formula, GI_*i *_is the GI value of food_*i *_from the GI tables [10]. CHO_*i *_is the total amount of carbohydrate in one serving of food_*i*_. F is the frequency of intake of the food_*i*_. A unit of GL is equivalent to 1 g of carbohydrate from white bread.

### Determination of metabolic syndrome

Participants meeting three or more of the Adult Treatment Panel III defining criteria for metabolic syndrome were regarded as having metabolic syndrome [2]. These criteria were: waist circumference >40 inches for men and >35 inches for women, triacylglycerol >150 mg/dL (>1.69 mmol/L) for both sexes, HDL-cholesterol <40 mg/dL (1.04 mmol/L) for men and <50 mg/dL (1.29 mmol/L) for women, blood pressure ≥ 130/85 mm Hg for both sexes, and fasting blood glucose ≥ 100 mg/dL (5.6 mmol/L) for both sexes. In addition, individuals that reported taking medications for blood pressure were counted as having blood pressure ≥ 130/85.

### Statistical analyses

SUDAAN statistical software (Windows, version 8.0.2; Research Triangle Institute, Research Triangle Park, NC) was used for statistical analysis in order to account for the complex survey design and in order to apply sampling weights. Use of sample weights in the data analysis takes differential probabilities of selection and non-coverage and non-response bias into consideration. The final examination weights were used in accordance with NHANES guidelines (least common denominator method). SAS (SAS for Windows, version 9.0, SAS Institute Inc, Cary, NC) and SPSS (SPSS for Windows, Chicago, IL, version 13.0) were also used for data management in conjunction with SUDAAN.

Participants' GL intake was adjusted for energy using the residual method [22]. Baseline characteristics of the participants were compared across quartiles of GL intake using analysis of variance for continuous variables and χ^2 ^test for categorical variables. Multivariate analysis of covariance (ANCOVA) was performed to determine the association between energy-adjusted GL and indices of metabolic syndrome for all subjects and separately for men and women. Multivariate-adjusted means were calculated for each component of metabolic syndrome for each quartile of GL intake. Standard errors were determined with Taylor Linearization method. In the regression models, GL was entered as the independent variable and each indicator of metabolic syndrome was entered as dependent variables. In the multivariate-adjusted ANCOVA, adjustments were made for sex, race-ethnicity, age, alcohol consumption, smoking status, BMI, prescription medication and supplement use, poverty income ratio, and intakes of protein, fat, and dietary fiber intake variables. Multiple comparisons were performed to determine difference between the means of GL quartiles for each indicator of metabolic syndrome with Bonferroni adjustment after testing the hypothesis with unpaired, two-tailed t-test.

Logistic regression was used to determine the multivariate-adjusted odds ratios (OR) and 95% confidence intervals (CI) for the presence of metabolic syndrome for each dietary GL quartile after adjusting the analysis for sex, age, race-ethnicity, BMI, smoking status, alcohol intake, prescription medicine and vitamin/mineral supplement use, poverty income ratio, and the intakes of dietary protein, fat and fiber. All values were considered statistically significant at *P *< 0.05.

## Results

The demographic characteristics of the study population are presented in Table 1. The study sample consisted of 3047 (58%) men and 1964 (42%) women. Individuals in the highest GL quartile were younger than those in the other GL quartiles (P < 0.001). More persons in the 1^st ^quartile GL group consumed vitamin/mineral supplements compared to those present in other groups. BMI differed within the GL categories (*P *= 0.013). There was a significant difference in BMI between the 1^st ^and 4^th ^quartiles of GL intake (26.6 vs. 25.9 kg/m^2^). Percentage of energy from fat was significantly higher in the 1^st ^GL quartile category (≅35%) compared to the 4^th ^quartile GL category (32.7%). Persons in the 4^th ^GL quartile consumed a largest percentage of energy from carbohydrate (*P *< 0.001). Total energy intake was also highest in those persons who consumed highest GL. The intake of dietary fiber was not associated with GL.

**Table 1:** Demographics, lifestyles, and health characteristics and dietary intakes of study population by intakes of glycemic load in the third National Health and Nutrition Examination Survey, 1988–1994^1^

	**Energy-adjusted glycemic load^2^**				
		
	**Quartile 1** **(< 119)**	**Quartile 2** **(119 – 157)**	**Quartile 3** **(157 – 204)**	**Quartile 4** **(≥ 204)**	***P *value^3^**
Glycemic load intake^4^					
Men	95	138	177	244	
Women	96	139	177	245	
Sex [*n *(%)]					<0.001
Men	659 (14.6)	688 (14.6)	714 (13.6)	986 (15.3)	
Women	443 (10.4)	458 (10.5)	506 (11.3)	557 (9.8)	
Race/ethnicity [*n *(%)]					<0.001
Non-Hispanic white	621 (49.6)	634 (50.6)	597 (47.6)	481 (38.4)	
Non-Hispanic black	411 (32.8)	312 (24.9)	288 (23)	288 (23)	
Mexican American	179 (14.3)	264 (21.1)	331 (26.4)	442 (35.3)	
Other	42 (3.4)	43 (3.4)	37 (3.0)	41 (3.3)	
Smokers [*n *(%)]	725 (57.9)	672 (53.6)	663 (52.9)	741 (59.2)	<0.001
Prescription medication users [*n *(%)]	429 (34.2)	436 (34.8)	435 (34.7)	398 (31.8)	<0.001
Vitamin/mineral supplement users [*n *(%)]	449 (35.8)	426 (34.0)	462 (36.9)	402 (32.1)	<0.001
Age^5 ^(*y*)	45.8 ± 0.5^a^	46.4 ± 0.5^a^	46.3 ± 0.5^a^	43.3 ± 0.5^b^	<0.001
Body mass index^5 ^(*kg/m*^2^)	26.6 ± 0.2^a^	26.4 ± 0.1^a,b^	26.4 ± 0.2^a,b^	25.9 ± 0.1^b^	0.013
Alcohol intake^5 ^(*drinks/mo*)	13 ± 0.8^a^	12 ± 0.6^a,b^	10 ± 0.5^b^	12 ± 0.7^a,b^	0.007
Dietary intakes^6^					
Total energy (*kcal/d*)	2290 ± 35^a,b^	2224 ± 3^a^	2229 ± 30^a^	2378 ± 33^b^	0.002
Fat (*g/d*)	92 ± 1.8^a^	86 ± 1.5 ^a, b^	85 ± 1.4 ^b, c^	89 ± 1.6^c^	<0.001
Protein (*g/d*)	88 ± 1.5^a^	83 ± 1.3^a,b^	84 ± 1.3^a,b^	88 ± 1.4^b^	<0.005
Carbohydrate (*g/d*)	254 ± 4.0^a^	258 ± 3.5^a^	269 ± 3.6^a^	295 ± 4.2^b^	<0.001
Fiber (*g/1000 kca/*)	7.7 ± 0.1	7.8 ± 0.1	8.1 ± 0.1	7.7 ± 0.1	0.077

^1 ^n = 5011 (men, 3047; women, 1964).

^2 ^Glycemic load is the product of the glycemic index of a food item and carbohydrate intake from that food in g divided by 100. Values not sharing common superscripts (a, b, c) are significantly different in a row using Bonferroni adjustment for multiple comparisons after testing the hypothesis with t-test.

^3 ^Significance for χ^2^-statistic for categorical variables or for *F*-statistic for continuous variables.

^4 ^Values are medians.

^5 ^Values are mean ± SEs.

^6 ^Data were derived from the 24-hour dietary recalls.

The association between energy-adjusted GL and the indicators of metabolic syndrome for all subjects and separately for men and women is shown in Table 2. In the multivariate-adjusted analysis, the GL was significantly associated with HDL-cholesterol in the combined sample (P = 0.007) and in men (*P *= 0.001) but not in women (*P *= 0.39). In men there was a significant difference in HDL-cholesterol concentrations between the 1^st ^and 3^rd ^GL quartiles groups (1.27 vs 1.16 mmol/L; *P *< 0.0001) and 1^st ^and 4^th ^GL quartiles groups (1.27 vs 1.15 mmol/L; *P *< 0.0001). GL was not associated with waist circumference, triacylglycerol, diastolic and systolic blood pressures, and fasting blood glucose in both men and women.

**Table 2:** Multivariate-adjusted components of metabolic syndrome according to the intakes of glycemic load in the third National Health and Nutrition Examination Survey, 1988–1994^1^

	**Energy-adjusted glycemic load^2^**				
		
	**Quartile 1** **(< 119)**	**Quartile 2** **(119 – 157)**	**Quartile 3** **(157 – 204)**	**Quartile 4** **(≥ 204)**	***P *value^3^**
Waist circumference (*cm*)					
All subjects	91 ± 0.2	91 ± 0.2	91 ± 0.2	91 ± 0.2	0.63
Men	94 ± 0.3	94 ± 0.3	95 ± 0.3	95 ± 0.2	0.19
Women	87 ± 0.4	87 ± 0.4	87 ± 0.3	87 ± 0.4	0.82
Triacylglycerol (*mmol/L*)					
All subjects	1.5 ± 0.04	1.6 ± 0.1	1.6 ± 0.04	1.6 ± 0.1	0.70
Men	1.7 ± 0.1	1.8 ± 0.1	1.7 ± 0.1	1.7 ± 0.1	0.60
Women	1.3 ± 0.1	1.3 ± 0.03	1.3 ± 0.04	1.4 ± 0.1	0.24
Systolic blood pressure (*mmHg*)					
All subjects	120 ± 0.6	120 ± 0.5	120 ± 0.5	120 ± 0.5	0.99
Men	124 ± 0.8	123 ± 0.6	123 ± 0.6	123 ± 0.7	0.69
Women	115 ± 0.8	117 ± 0.7	116 ± 1.0	117 ± 0.7	0.46
Diastolic blood pressure (*mmHg*)					
All subjects	74 ± 0.4	74 ± 0.4	74 ± 0.4	74 ± 0.4	0.73
Men	77 ± 0.6	76 ± 0.5	76 ± 0.5	76 ± 0.5	0.54
Women	71 ± 0.5	72 ± 0.5	71 ± 0.6	71 ± 0.4	0.77
Fasting plasma glucose (*mmol/L*)					
All subjects	5.2 ± 0.04	5.2 ± 0.03	5.2 ± 0.02	5.2 ± 0.02	0.46
Men	5.4 ± 0.04	5.3 ± 0.04	5.3 ± 0.03	5.4 ± 0.03	0.28
Women	5.1 ± 0.04	5.1 ± 0.04	5.1 ± 0.1	5.0 ± 0.3	0.65
HDL cholesterol (*mmol/L*)					
All subjects	1.32 ± 0.02^a^	1.30 ± 0.01^a,b^	1.28 ± 0.01^a,b^	1.25 ± 0.02^b^	0.007
Men	1.27 ± 0.02^a^	1.20 ± 0.02^a,b^	1.16 ± 0.02^b^	1.15 ± 0.02^b^	0.0001
Women	1.40 ± 0.2	1.43 ± 0.02	1.43 ± 0.02	1.39 ± 0.03	0.39

^1 ^n = 5011 (men, 3047; women, 1964). Glycemic load is the product of the glycemic index of a food item and carbohydrate intake from that food in g divided by 100. Metabolic syndrome was defined according to the Adult Treatment Panel III Guidelines (waist circumference >40 inches for men and >35 inches for women, triacylglycerol <150 mg/dL (1.69 mmol/L) for both sexes, HDL-cholesterol <40 mg/dL (1.04 mmol/L) for men and <50 mg/dL (1.29 mmol/L)for women, blood pressure ≥ 130/85 mm Hg for both sexes, and fasting blood glucose ≥ 100 mg/dL (5.6 mmol/L) for both sexes.

^2 ^Values are mean ± SEs. Analysis of covariance (ANCOVA) with energy-adjusted glycemic load as the independent variable and indicators of metabolic syndrome as dependent variables. Values not sharing common superscript (a, b) are significantly different from each other within the indicator of metabolic syndrome (across row) using Bonferonni adjustment for multiple comparisons after testing the hypothesis with unpaired, two-tailed *t*-test. Analysis was adjusted for age, race-ethnicity, smoking status, poverty income ratio, prescription medication use, vitamin/mineral supplement use, and intakes of alcohol, protein, fat, and dietary fiber.

^3 ^Significance of metabolic syndrome indicator variable in the analysis of covariance (*P *for Wald *F*).

The association between energy-adjusted GL and prevalence of metabolic syndrome is presented in Table 3. We found no association between energy-adjusted GL and prevalence of metabolic syndrome in the sex-race-ethnicity-age-adjusted (*P *for trend < 0.21) or in the multivariate-adjusted logistic regression analysis (*P *for trend < 0.09).

**Table 3:** Multivariate-adjusted odds ratio (OR) and 95% confidence intervals (CI) for metabolic syndrome according to intakes of glycemic load in the third National Health and Nutrition Examination Survey, 1988–1994^1^

	**Energy-adjusted glycemic load^2^**				
		
	**Quartile 1^3^** **(< 119)**	**Quartile 2** **(119 – 157)**	**Quartile 3** **(157 – 204)**	**Quartile 4** **(≥ 204)**	***P *value^4^**
Positive for metabolic syndrome, n (%)	212 (4.2)	218 (4.4)	239 (4.8)	274 (5.5)	
Age, sex, and race-ethnicity adjusted	1.00	0.99 (0.75, 1.29)	1.22 (0.91, 1.63)	0.83 (0.62, 1.10)	0.21
Multivariate-adjusted^5^	1.00	0.96 (0.61, 1.50)	1.37 (0.88, 2.12)	0.81 (0.53, 1.23)	0.09

^1 ^n = 5011 (men, 3047, women, 1964). Metabolic syndrome was defined according to the Adult Treatment Panel III Guidelines (waist circumference >40 inches for men and >35 inches for women, triacylglycerol <150 mg/dL (1.69 mmol/L) for both sexes, HDL-cholesterol <40 mg/dL (1.04 mmol/L) for men and <50 mg/dL (1.29 mmol/L) for women, blood pressure ≥ 130/85 mm Hg for both sexes, and fasting blood glucose ≥ 100 mg/dL (5.6 mmol/L) for both sexes.

^2 ^Glycemic load is the product of the glycemic index of a food item and carbohydrate intake from that food in g divided by 100.

^3 ^Referent category.

^4 ^Significance of metabolic syndrome indicator variable in the multivariate logistic regression analysis (*P *for Wald *F*).

^5 ^Logistic regression was adjusted for sex, age, race-ethnicity, smoking status, poverty income ratio, prescription medication use, vitamin/mineral supplement use, and intakes of alcohol, protein, fat, and dietary fiber.

## Discussion

To our knowledge this is the first study that reports the association between GL and all components of metabolic syndrome using the data from a nationally representative sample survey of US residents. We found that in all subjects and in men but not in women, high GL was associated with low HDL-concentrations in the multivariate-adjusted analysis after taking several confounding variables into consideration. Also, no association was observed between energy-adjusted GL and prevalence of metabolic syndrome in either sex. Previous studies on the association between GL and some indicators of metabolic syndrome yielded equivocal observations. The present observation of inverse relation between GL and HDL-cholesterol is in agreement with the observation made by Ford and Liu [20]. They reported a decrease in HDL with increasing GL (*P *< 0.001). However, they [20] did not report the association between GL and other indicators of metabolic syndrome. Later, Liu et al [23] reported that GL was inversely associated with HDL-cholesterol in 280 postmenopausal women (*P *= 0.03). In a Dutch population, Du et al [24] found no association between GL and several metabolic components of metabolic syndrome (fasting glucose, HDL-cholesterol, and triacylglycerol). Recently, Kim et al [25] reported a positive relation between low HDL-cholesterol and high GL and an increased risk of developing the metabolic syndrome in those with the highest quintile of GL intake compared with those with the lowest quintile of GL intake in Korean women with a BMI ≥ 25 kg/m^2^.

In this study, it is not known why there was a difference between men and women with regard to the association between GL and HDL-cholesterol. Estrogen has a protective effect on lipid profile [26]. Kaim-Karakas et al [27] reported that post-menopausal women responded differently to low-fat diets. The mean age of the women in our sample was 43 ± 0.4 y. This would classify many as pre-menopausal [28]. Thus, the difference in association between GL and HDL-cholesterol between sexes may be due to hormonal differences between men and women. In women, lack of association in this study and an inverse association in Liu et al's study [23] between GL and HDL-cholesterol can be attributed to the differences in characteristics of subjects.

High GL diets have been associated with increased fasting triacylglycerol concentrations due to the potential accumulation of atherogenic triacylglycerol-rich VLDL remnants [23,29,30]. This has been attributed to an increase in carbohydrate as a caloric compensation for dietary fat [31]. Previous investigations on the effect of carbohydrate on the lipid profile have been conducted in individuals who consumed < 20% energy from total fat [32]. Whereas, in this study the energy intake from fat was 33–35%. However, not all reports have shown that consumption of low-fat, high carbohydrate diets leads to unfavorable metabolic profile [32]. Conflicting results between studies can be attributed to the differences between characteristics of subjects and procedures used in the data analysis.

No association was observed between fasting blood glucose and energy-adjusted GL in this study. Others reported that diets high in GL increase the risk of impaired glucose tolerance [33]. Individuals with diabetes were excluded from the current study. Thus, the individuals with metabolic syndrome were those with impaired fasting glucose. These individuals are at high risk of developing type-2 diabetes [34]. Previously, investigators noted a reduced risk of developing type-2 diabetes in those consuming a low GL, high cereal fiber diet in the Nurses' Health and Health Professionals' Follow-Up study [9]. In contrast, there was no association between GL or GI and the risk of type-2 diabetes in the Iowa Women's Health Study [35].

There is only one published study on the association between GL and metabolic syndrome [14]. In the Framingham Cohort, there was no association between GL and the prevalence of metabolic syndrome. This is consistent with our findings. McKeown et al [14] observed a relation between GI and the prevalence of metabolic syndrome among the predominantly white sample. Among individuals in the highest quintile of GI intake, there was a 41% increased risk of metabolic syndrome. The authors noted that individuals in the highest quintile of whole-grains, dietary fiber, cereal fiber, and fruit fiber with a low GI had a decreased risk of insulin resistance. There was a 38% reduction in the prevalence of metabolic syndrome for the highest quintile of cereal fiber and a 33% reduction in the prevalence of metabolic syndrome for whole-grains [14].

Although the determination of GL based on food frequency intake data has been reported earlier [20,21], it is possible that the food frequency data may not reflect the number of servings actually consumed. Noethlings et al [36] reported that data on portion sizes add little to the variance in food intake and the major part of variance in food intake is explained by the frequency of food consumption alone. Cereal fiber has previously been shown to drive the relationship with GL [9]; however, this variable was not measured in the NHANES III. Alternatively, we have adjusted the analysis for total dietary fiber intake. A shortcoming of the current GI tables is the lack of available values for all food items commonly consumed. When published values for GI were not available, an appropriate substitution was made. It is possible that such a substitution may have led to misclassification of the participants' intake and overall average GL. Because of cross-sectional nature of this study, cause and effect measurement is not possible.

There are no specific dietary recommendations exist for the treatment and prevention of metabolic syndrome. A treatment goal is to manage the individual components of metabolic syndrome in order to prevent or delay the development of CVD or type-2 diabetes [2]. Strategies for the management of metabolic syndrome are weight management and physical activity. Weight control improves all components of metabolic syndrome [13]. A dietary recommendation for metabolic syndrome is incorporation of foods that minimize fluctuations in blood glucose [37]. In general, diets low in GL might be beneficial in maintaining healthy lipid profile. Until more evidence is available on the role of GI and GL in metabolic syndrome, it is prudent to follow the dietary guidelines and prepare foods with little added sugar, and consume a minimum of three servings of whole grains/day [38].

## Competing interests

The authors declare that they have no competing interests.

## Authors' contributions

Culberson and Ganji were responsible for study design and drafting the manuscript. Culberson and Kafai were responsible for data collection, data management, and statistical analyses. Kafai, Ganji, and Culberson were responsible for interpretation of data and revision of manuscript. At the time of the study, Culberson was a graduate student and Ganji was a faculty member in the Departments of Clinical Nutrition and Food and Nutrition, Rush University Medical Center, Chicago. The data presented in this manuscript come from Culberson's Master's thesis and she acknowledges her thesis committee. All authors read and approved the final manuscript.

## References

[B1] Ford ES, Giles WH, Mokdad AH (2004). Increasing prevalence of the metabolic syndrome among US adults. Diabetes Care.

[B2] Third Report of the National Cholesterol Education Program (NCEP) Expert Panel on Detection, Evaluation, and Treatment of High Blood Cholesterol in Adults (Adult Treatment Panel III) Full Report. National, Heart, Lung, and Blood Institute Home Page.

[B3] Ludwig DS, Majzoub JA, Al-Zahrani A, Dallal GE, Blanco I, Roberts SB (1999). High glycemic index foods, overeating, and obesity. Pediatrics.

[B4] Gross LS, Li L, Ford ES, Liu S (2004). Increased consumption of refined carbohydrate and the epidemic of type 2 diabetes in the United States: an ecologic assessment. Am J Clin Nutr.

[B5] Garg A, Bantel JP, Henry RR, Coulston AM, Griver KA, Raatz SK, Brinkley L, Chen YD, Grundy SM, Huet BA (1999). Effects of varying carbohydrate content of diet in patients with noninsulin dependent diabetes mellitus. JAMA.

[B6] Clark R, Frost C, Collins R, Appleby P, Peto R (1997). Dietary lipids and blood cholesterol: quantitative meta-analysis of metabolic ward studies. Br J Nutr.

[B7] Riccardi G, Rivellese AA (2000). Dietary treatment of the metabolic syndrome – the optimal diet. Br J Clin Nutr.

[B8] Jenkins DJ, Wolever TM, Taylor RH, Barker H, Fielden H, Baldwin JM, Bowling AC, Newman HC, Jenkins AL, Goff DV (1981). Glycemic index of foods: a physiological basis for carbohydrate exchange. Am J Clin Nutr.

[B9] Salmeron J, Manson JE, Stampfer MJ, Colditz GA, Wing AL, Willett WC (1997). Dietary fiber, glycemic load, and risk of non-insulin-dependent diabetes mellitus in women. JAMA.

[B10] Foster-Powell K, Holt SH, Brand-Miller JC (2002). International table of glycemic index and glycemic load values: 2002. Am J Clin Nutr.

[B11] Haffner SM, Valdez RA, Hazuda HP, Mitchell BD, Morales PA, Stern MP (1992). Prospective analysis of the insulin-resistance syndrome (syndrome X). Diabetes.

[B12] Isomaa B, Henricsson M, Almgren P, Tuomi T, Taskinen MR, Groop L (2001). The metabolic syndrome influences the risk of chronic complications in patients with type II diabetes. Diabetologia.

[B13] Liu S, Willett WC, Stampfer MJ, Hu FB, Franz M, Sampson L, Hennekens CH, Manson JE (2000). A prospective study of dietary glycemic load, carbohydrate intake, and risk of coronary heart disease in US women. Am J Clin Nutr.

[B14] McKeown N, Meigs JB, Liu S, Saltzman E, Wilson PW, Jacques P (2004). Carbohydrate nutrition, insulin resistance, and the prevalence of the metabolic syndrome in the Framingham Offspring Cohort. Diabetes Care.

[B15] Grundy SM, Brewer HB, Cleeman JI, Smith SC, Lenfant C (2004). Definition of metabolic syndrome: Report of the National Heart, Lung, and Blood Institute/American Heart Association conference on scientific issues related to definition. Circulation.

[B16] National Center for Health Statistics (1996). Third National Health and Nutrition Examination Survey, 1988–1994, NHANES III reference manuals and reports. Public use data files Centers for Disease Control and Prevention Home Page.

[B17] National Center for Health Statistics (1996). Third National Health and Nutrition Examination Survey, 1988–1994; NHANES III examination data file. Public use data files Centers for Disease Control and Prevention Home Page.

[B18] Frohlich ED (1988). Recommendations for blood pressure determination by sphygmomanometry. Ann Intern Med 1988, 109:612.

[B19] Agriculture Research Service Survey nutrient data bases for NHANES III, Phase 1 and Phase 2.

[B20] Ford ES, Liu S (2001). Glycemic index and serum high-density lipoprotein cholesterol concentration among US adults. Arch Intern Med.

[B21] Agriculture Research Service (2003). US Department of Agriculture. National Nutrient Database for Standard Reference release 16.

[B22] Willet WC, Howe GR, Kushi LH (1997). Adjustment for total energy intake in epidemiologic studies. Am J Clin Nutr.

[B23] Liu S, Manson JE, Stampfer MJ, Holmes MD, Hu FB, Hankinson SE, Willett WC (2001). Dietary glycemic load assessed by food-frequency questionnaire in relation to plasma high-density lipoprotein cholesterol and fasting plasma triacylglycerols in postmenopausal women. Am J Clin Nutr.

[B24] Du H, van Der DLA, van Bakel MME, Kallen CJH van der, Blaak EE, van Greevenbroek MMJ, Jansen EHJM, Nijpels G, Stehouwer CDA, Dekker JM, Feskens EJM (2008). Glycemic index and glycemic load in relatin to food and nutrient intake and metabolic risk factors in a Dutch population. Am J Clin Nutr.

[B25] Kim K, Yun SH, Choi BY, Kim MK (2008). Cross-sectional relationship between dietary carbohydrate, glycemic index, glycemic load and risk of the metabolic syndrome in a Korean population. Br J Nutr.

[B26] Mosca L (2002). Optimal management of cholesterol levels and prevention of coronary heart disease in women. Am Fam Physician.

[B27] Kaim-Karakas S, Almario RU, Mueller WM (2000). Changes in plasma lipoprotein during low-fat, high carbohydrate diets: effects of energy intake. Am J Clin Nutr.

[B28] Cramer DW, Xu H (1996). Predicting age at menopause. Maturitas.

[B29] Fried SK, Rao SP (2003). Sugars, hypertriglyceridemia, and cardiovascular disease. Am J Clin Nutr.

[B30] Hellerstein MK (2002). Carbohydrate-induced hypertriglyceridemia: modifying factors and implications for cardiovascular risk. Curr Opin Lipidol.

[B31] Parks E (2001). Effect of dietary carbohydrate on triglyceride metabolism in humans. J Nutr.

[B32] Barnard R, Jung T, Inkeles SB (1994). Diet and exercise in the treatment of NIDDM – the need for early emphasis. Diabetes Care.

[B33] Ludwig DS (2002). The glycemic index: physiological mechanism relating to obesity, diabetes, and cardiovascular disease. JAMA.

[B34] The Expert Committee on the Diagnosis and Classification of Diabetes Mellitus (2003). Follow-up report on the diagnosis of diabetes mellitus. Diabetes Care.

[B35] Meyer K, Kushi L, Jacobs D (2000). Carbohydrates, dietary fiber, and incident type two diabetes in older women. Am J Clin Nutr.

[B36] Noethlings U, Hoffmann K, Bergmann MM, Boeing H (2003). Portion size adds limited information on variance in food intake of participants in the EPIC-Postdam study. J Nutr.

[B37] Miller JC (1994). Importance of glycemic index in diabetes. Am J Clin Nutr.

[B38] US Dept of Agriculture (2005). Nutrition and your health: Dietary Guidelines for Americans.

